# Eukaryotic gene invasion by a bacterial mobile insertion sequence element IS2 during cloning into a plasmid vector

**DOI:** 10.1186/2041-9414-1-2

**Published:** 2010-05-26

**Authors:** Alireza G Senejani, Joann B Sweasy

**Affiliations:** 1 Department of Therapeutic Radiology and Human Genetics, Yale University School of Medicine, New Haven, CT 06520, USA

## Abstract

*Escherichia coli (E. coli) *are commonly used as hosts for DNA cloning and sequencing. Upon transformation of *E. coli *with recombined vector carrying a gene of interest, the bacteria multiply the gene of interest while maintaining the integrity of its content. During the subcloning of a mouse genomic fragment into a plasmid vector, we noticed that the size of the insert increased significantly upon replication in *E. coli*. The sequence of the insert was determined and found to contain a novel DNA sequence within the mouse genomic insert. A BLAST search of GenBank revealed the novel sequence to be that of the Insertion Sequence 2 (IS2) element from *E. coli *that was likely inserted during replication in that organism. Importantly, a detailed search of GenBank shows that the IS2 is present within many eukaryotic nucleotide sequences, and in many cases, has been annotated as being part of the protein. The results of this study suggest that one must perform additional careful analysis of the sequence results using BLAST comparisons, and further verification of gene annotation before submission into the GenBank.

## Findings

In October 2009, GenBank (the NIH database in Bethesda, Maryland U.S.A.) reported the genetic sequence database exceeded 106 billion nucleotide bases in more than 3,000, 000 named organisms [1]. GenBank, along with the European Molecular Biology Laboratory (EMBL-Bank in Hinxton, U.K.), and the DNA Data Bank of Japan (Mishima, Japan) are the three members of the International Nucleotide Sequence Database Collaboration that exchange information daily to ensure a consistent and complete collection of nucleotide sequence information. This database is expected to keep growing exponentially as sequencing is becoming economically more affordable and demand increases. Therefore, to ensure greater integrity of the data appearing in GenBank, further constructive steps to verify the identity of sequences before submission is becoming increasingly important.

Much of the information in GenBank was obtained by first subcloning a fragment of DNA, followed by its amplification and sequencing. During this process *Escherichia coli (E. coli) *are commonly used as hosts to multiply the gene of interest faithfully. In this study we report how unusual gene invasions during gene cloning in *E. coli *have caused incorrect annotation of a number of genes and proteins from numerous diverse species.

**Figure 1:**
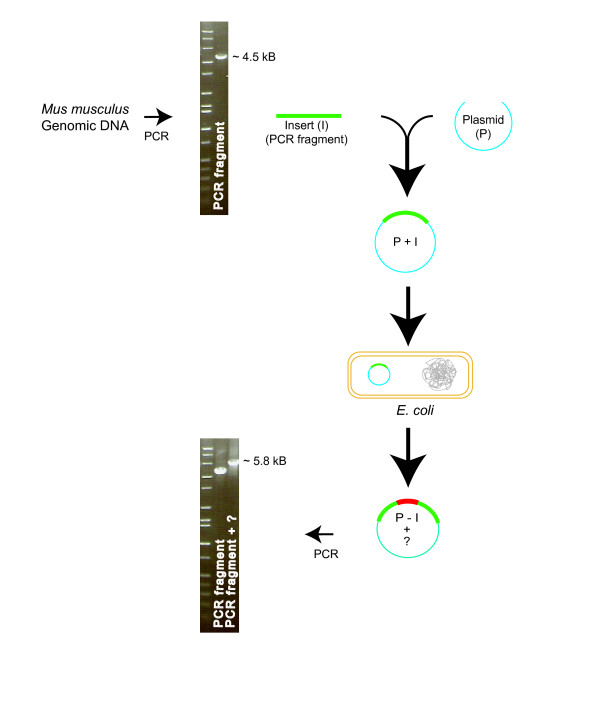
**Schematic diagram illustrating the cloning process that lead to detection of an extra DNA element integrated into the insert during the process**. A PCR fragment, amplified from the gene of interest, with a size of about 4.5 kb, was inserted into a plasmid. The recombinant plasmid was then transformed into *E. coli*. The PCR was performed using the same sets of primers and extracted recombinant plasmids as a template. The resulting PCR fragment appeared to be about 5.8 kb long. This indicates the presence of extra DNA inside the insert. Further multiple restriction digestion analyses and sequencing confirmed the presence of the extra 1.3 kb DNA fragment within the insert.

We are using a gene targeting approach to generate various knock-in mice. Using PCR, a fragment of the mouse genomic DNA that is 4.5 kb in length and harbors the region of interest was amplified, as shown in Figure 1. After digestion with appropriate restriction enzymes the fragment was inserted into a plasmid. During screening of the transformed host, *E. coli *Xl1-Blue (Stratagene), one of the resulting recombinant plasmids appeared to carry the insert. However, results from multiple restriction digestions and PCR analysis suggested the presence of an extra fragment of DNA within the insert (Figure 1). After sequencing and comparison of the extra DNA fragment with the entire sequence of the cloned PCR product, the results indicated that the extra piece of DNA element, 1.3 kb in length, was not a result of a duplication of any region of the original PCR amplified region. Furthermore, since the *E. coli *Xl1-Blue host was recombination deficient, it was not expected to occur as a consequence of potential recombination between the insert and other plasmids or the host genomic DNA.

**Figure 2:**
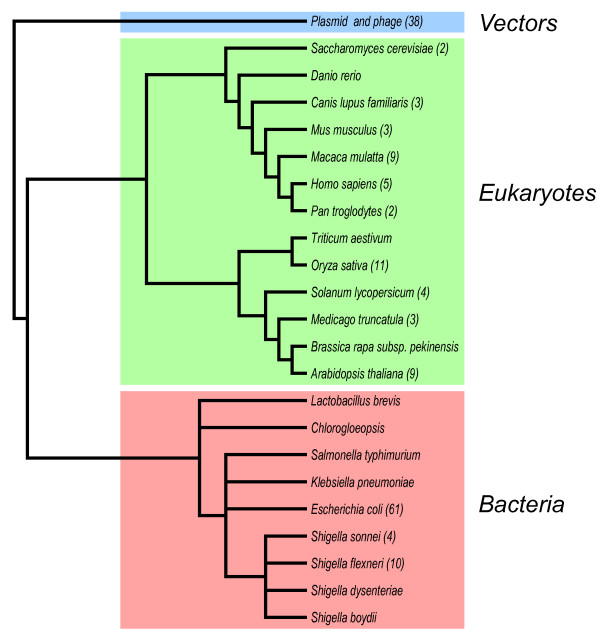
**Taxonomy BLAST reports of species submitted into the GenBank that contain the bacterial insertion element IS2 **[1,8]. The IS2 elements in each of these organisms is nearly identical. The numbers indicate how often the insertion element IS2 was found in the BLAST hitlist.

Astonishingly, using nucleotide-nucleotide BLAST and the extra 1.3 kb DNA as a query sequence against the DNA database, we learned that the presence of the unidentified DNA has been reported in many diverse species, as shown in Figure 2. Among the organisms reported to have the identical DNA fragment are members of eukaryotes and bacteria domains. In bacteria, *E. coli *received the highest number of hits (61); this was followed by *Shigella *with more than 10 hits. Among eukaryotes, *Oryza sativa *received the highest number of hits, 11 times; this was followed by *Arabidopsis thaliana, Macaca mulatta, and Homo sapiens *with 9, 9, and 5 hits, respectively (see Figure 2).

Results from further sequence analysis indicated that the extra DNA was the *E. coli *insertion sequence element IS2 that likely incorporated itself into the insert during the cloning process. IS2 is a short 1.3 kb DNA sequence [2] that acts like a simple self mobile genetic element [3]. Although the insertion sequence elements often act as genomic parasites they can sometimes cause chromosome rearrangements and produce mutations leading to elimination or adaptation of their host organism [4]. Multiple copy presence of the IS2 was reported in *E. coli *more than thirty years ago [5]. During the integration of IS2 into the 4.5 kb fragment we used in our studies, a short piece of DNA with a size of six nucleotides (AGAAAG) was duplicated at the end of insertion. We noticed the presence of a similar duplication (5-8 nucleotides) in nearly all of other entries reported into the GeneBank where the presence of IS2 was recognized (see Table 1). As shown in Table 1, there are 11 copies of IS2 that were detected when the IS2 nucleotide sequence was used to search against the entire genome of the *E. coli *W311 [GenBank: AP009048]. With an exception of one (AACCC), which was also reported in an earlier study [6], there is no detectable similarity between duplicated regions found in the 11 copies of IS2 in *E. coli*.

**Table 1:** List of some selected genes that contain the IS2 element.

Gene name and ID	Location^a^	Surrounding IS2 sequences^b^
dbj|AP009048.1| *Escherichia coli *W3110, complete genome, Length = 4646332	3742928-3744271 2320789-2322130 381819-380478 2071073-2072413 4504197-4502855 1105615-1106957 1302369-1301034 1469618-1470959 2995017-2996347 3186087-3184747 1653272-1652545	GAAAT**TGG...TCT**AGAAATTGG GATCG**TGG...TCT**AGATCGT GTAAT**TGG...TCT**AGTAATT GTGGC**TGG...TCT**AGTGGC ACAAGG**TGG...TCT**ACAAGG AACCCT**TGT...TCT**AACCCT TAATATC**TGT...TCT**AATATC GAACCC**TGT...TCT**AAACCC tttat**TGG...TCT**aaacttg ATAAC**TGG...TCT**AATAAC acctg**TGG...T**tcgtc
dbj|AP008210.1| *Oryza sativa*, chromosome 4, Length = 35498469	27366872-27368214 11718374-11719715 13525525-13524204	AGAAAG**TGG...TCT**AGAAAG GTTAG**TGG...TCT**AGTTAGT AGAGAT**TGG...TCT**AGAGATT
emb|AL731613.5|OSJN00257*Oryza sativa*, chromosome 4, Length = 133967	18027-19368	AGAAAG**TGG**...**TCT**AGAAAG
gb|AC004776.1| *Homo sapiens*, chromosome 5, Length = 89626	20138-18796	ATTTCC**TGG...TCT**ATTTCCT
gb|AC018501.9| *Homo sapiens*, chromosome 3, Length = 202070	72232-70890	TATCTGG**TGG...TCT**ATCTGG
gb|AC170856.2| *Medicago truncatula*, chromosome 2, Length = 101215	69921-68580	TCAAG**TGG...TCT**ATCAAGT
gb|AC191971.14| *Rhesus Macaque*, genomic DNA, Length = 174949	169869-171210	ACACAG**TGG...TCT**ACACAG
gb|AC202613.6| *Rhesus macaque*, genomic DNA, Length = 181783	146766-145426	GTTCC**TGG...TCT**AGTTCC
gb|AC200594.3| *Rhesus macaque*, genomic DNA, Length = 171745	99933-98589	TAGGTGTT**TGG...TCT**AGTGTTT
gb|AC198308.7| *Rhesus Macaque*, genomic DNA, Length = 143534	76175-74834	GTTTG**TGG...TCT**AGTTTGT
gb|AC196863.3| *Macaca mulatta*, chromosome 2, Length = 172779	155658-154318	CAAAC**TGG...TCT**ACAAAC
gb|AC193812.4| *Canis Familiaris*, chromosome 13, Length = 196541	31412-30070	TAGATCT**TGG...TCT**AGATCT
gb|AC187015.8| *Canis familiaris*, chromosome 33, Length = 215278	178002-176660	TAGTTGG**TGG...TCT**AGTTGG
dbj|AK229126.1| *Arabidopsis thaliana*, cDNA, Length = 4564	2374-3716	AAAGAG**TGG...TCT**AAAGAGT
dbj|AK229400.1| *Arabidopsis thaliana*, cDNA, Length = 3239	869-2210	AGAAGG**TGG...TCT**AGAAGG
gb|AY064986.1| *Arabidopsis thaliana*, cDNA, Length = 3309	1180-2523	TAGAAGG**TGG...TCT**AGAAGGT
dbj|AK226701.1| *Arabidopsis thaliana*, cDNA, Length = 5499	3369-2028	AGAATT**TGG...TCT**AGAATT
gb|AC193907.3| *Pan troglodytes*, chromosome x, Length = 161221	90423-91765	AGCAGG**TGG...TCT**AGCAGGT

^a^The location and the duplicated nucleotides ^b^surrounding IS2 sequences are shown. The underlined sequences are the first and last triplet nucleotides of the IS2 element.

We have observed a phenomenon in the laboratory whereby bacterial IS2 elements are incorporated into eukaryotic genes during amplification of a recombinant plasmid vector transformed into *E. coli *cells. The presence of the bacterial IS2 element is reported in many complete/partial genomic DNA or cDNA sequences of numerous diverse eukaryotic species including *Homo sapiens, Mus musculus, Macaca mulatta, Oryza sativa, Arabidopsis thaliana*, and many others submitted into GenBank. The insertion of the IS2 element occurred most likely during replication in *E. coli*, similar to our study. For example, if you BLAST the IS2 element from *E. coli *K-12, one of the top hits is GenBank: AK227066.1*Arabidopsis thaliana *mRNA for calcium-dependent protein kinase 19 (CDPK19). If you then take the this *Arabidopsis *sequence and do a blastn, the top three hits are the *Arabidopsis *CDPK19 that DO NOT contain the IS2 [GI: 145361922, 30687319, and 836941], whereas the next hits belong to IS2 elements in a variety of organisms including *E. coli*, plants and animals. On some occasions the IS2 DNA sequence is unintentionally claimed to be a host protein or a part of host protein coding region; i.e. [GenBank: BAE99182] from *Arabidopsis thaliana *or [GenBank: CAI64485] from *Oryza stativarotein *[7]. Similarly, when the nucleotide sequence of IS1 was used as a query, the results indicated the presence of this genetic mobile element in many genes found in numerous members of Eukaryota (data not shown). The results of this study suggest one must perform additional careful analysis of the BLAST results from cloned sequences, and further verification of gene annotation before submission into GenBank.

## Competing interests

The authors declare that they have no competing interests.

## Authors' contributions

AS carried out the molecular genetic studies, participated in the sequence alignment and drafted the manuscript. JS coordinated, helped to design of the study and improved the manuscript. All authors read and approved the final manuscript.
